# Diversity and function of tumor-associated macrophages in brain metastases: mechanisms and therapeutic prospects

**DOI:** 10.3389/fimmu.2026.1756299

**Published:** 2026-02-05

**Authors:** Yingping Ma, Hongyu Wang, Xinman Dou, Qiang Li

**Affiliations:** 1 Department of Neurosurgery, The Second Hospital of Lanzhou University, Lanzhou, China; 2 Nursing Department, The Second Hospital of Lanzhou University (The Second Clinical Medical College), Lanzhou, China

**Keywords:** brain metastasis, immunosuppression, immunotherapy, tumor microenvironment, tumor-associated macrophages

## Abstract

Brain metastasis significantly worsens prognosis in late-stage cancer., with Its treatment hindered by the blood-brain barrier (BBB) and an immunosuppressive tumor microenvironment. Within this environment, tumor-associated macrophages (TAMs) represent the predominant immune population. Through their roles in immune modulation, angiogenesis, and tumor invasion, TAMs are critical drivers of disease progression. TAMs are highly heterogeneous. While traditionally categorized into M1 (anti-tumor) or M2 (pro-tumor) phenotypes, this dichotomy is an oversimplification. Recent single-cell studies have revealed a spectrum of functional subpopulations, such as lipid-associated, interferon-responsive, and pro-angiogenic TAMs, with M2-like states typically prevailing to mediate immunosuppression. This review explores the diversity and functions of TAMs in brain metastasis. We first detail their biological characteristics, including origins, heterogeneous subtype classifications (e.g., lipid-associated macrophages that extend beyond the simple M1/M2 dichotomy), and polarization states. We further discuss how polarization is regulated by signaling pathways (e.g., STAT, NF-κB) and microenvironmental factors (e.g., hypoxia, metabolic reprogramming). We examine TAM roles from pre-metastatic niche formation to tumor colonization, using breast and lung cancer brain metastases to illustrate how TAMs disrupt the BBB and facilitate immune evasion through molecules like ANGPTL4 (angiopoietin-like 4) and MMP9. Key pathways of TAM-tumor cell interactions, including neuro-cancer interactions, immune-metabolic regulation, and exosome-mediated communication, are also discussed. Targeting TAMs offers promising therapeutic avenues. These strategies include reprogramming TAMs (e.g., using CSF1R inhibitors), combining TAM-targeted therapy with immune checkpoint inhibitors, and developing novel approaches such as nanotechnology and CAR-macrophages. However, several challenges remain, including TAM heterogeneity, lack of targeting specificity, and the obstacle of BBB delivery. Future research should leverage technologies like single-cell sequencing and spatial transcriptomics to decode TAM heterogeneity, and develop personalized treatments based on biomarkers such as GPNMB and TRAIL, aiming to improve patient outcomes in brain metastasis.

## 1 Introduction

**Figure 1:**
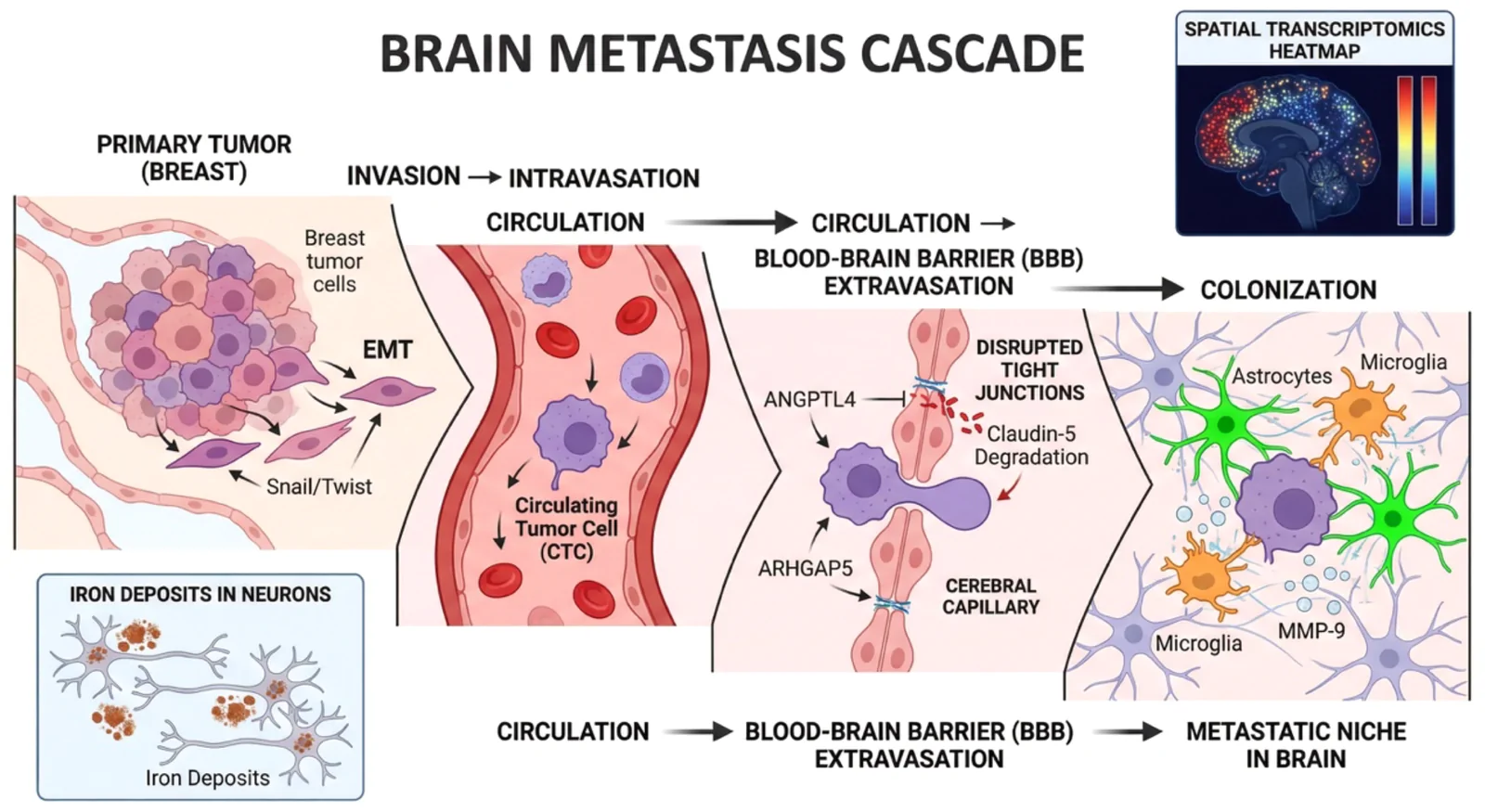
Mechanistic cascade of breast cancer brain metastasis.

Brain metastasis is one of the most common complications in the late stages of cancer, particularly in patients with solid tumors such as breast cancer, lung cancer, and melanoma (**Figure 1**). Its incidence is increasing, and the prognosis remains extremely poor (1). According to literature reports, the median overall survival of patients with brain metastases is low, highlighting the urgency and challenges of clinical treatment (2, 3). Significant obstacles to treatment include the physical and physiological barrier functions of the blood-brain barrier (BBB) and the immunosuppressive characteristics of the tumor microenvironment (TME) (4). The BBB restricts the intracranial delivery of most chemotherapeutic drugs and immunotherapeutic agents, while the immunosuppressive microenvironment promotes the survival, proliferation, and escape of tumor cells through various mechanisms (4). Consequently, the efficacy of systemic immunotherapies, particularly immune checkpoint inhibitors (ICIs), remains limited in patients with brain metastases, underscoring the urgent need for novel therapeutic strategies that can overcome this immunosuppressive barrier.

In the TME of brain metastases, tumor-associated macrophages (TAMs) are the most abundant immune cell population, comprising up to 50% of all immune cells. They play a central role in regulating immune responses and tumor progression (5). TAMs originate from either resident brain microglia or peripherally recruited monocytes. By secreting cytokines, and chemokines, and remodeling the extracellular matrix, they directly promote tumor cell invasion, angiogenesis, and metastasis (6). For instance, in glioblastoma (GBM), TAMs inhibit T-cell function, leading to immune evasion and treatment resistance by expressing immune checkpoint molecules such as PD-L1 and secreting immunosuppressive factors like including IL-10 and TGF-β (7). Furthermore, the heterogeneity of TAMs, such as the M1 and M2 subtypes, complicates their functions. M2-like TAMs are typically associated with a pro-tumor phenotype and predict poor prognosis (7).

Epidemiological data on brain metastases indicate that up to 40% of cancer patients will develop brain metastases. With improvements in primary tumor treatment, the incidence of brain metastases may further increase (8). The formation of an immunosuppressive microenvironment involves various cellular and molecular mechanisms, including the recruitment and polarization of TAMs, cellular metabolic reprogramming, and neuro-immune interactions (4). For example, research in cancer neuroscience has shown that neuronal activity can influence TAM function by secreting factors such as brain-derived neurotrophic factor (BDNF) and glutamate, thereby enhancing the colonization of brain metastases (9). Therefore, understanding the role of TAMs in brain metastases not only helps to elucidate the mechanisms of the disease but also provides critical targets for developing novel immunotherapeutic strategies. In summary, the challenge of brain metastases stems from its unique microenvironmental characteristics, with TAMs being the dominant immune cells in the TME, playing a crucial role in driving immunosuppression and therapeutic resistance.

This review first elucidates the biological characteristics of TAMs, including their origins, subtypes, and polarization. Subsequently, we summarize the role of TAMs in brain metastases, from the formation of pre-metastatic niches to colonization. Next, we analyze the key signaling pathways underlying interactions between TAMs and tumor cells. Finally, we compile therapeutic strategies targeting TAMs, ranging from preclinical studies to clinical translation, and summarize future challenges and directions. We aim to provide a reference for future research on TAMs in brain metastases. Furthermore, this review will emphasize the critical functional and phenotypic heterogeneity of TAMs, which is shaped not only by their polarization states but also, uniquely within the brain, by their cellular origin—resident microglia versus peripherally recruited macrophages—going beyond the conventional M1/M2 dichotomy.

## 2 The brain’s immune microenvironment and anatomical landscape as a metastatic niche

To understand the unique pathophysiology of brain metastasis, it is essential first to grasp the Central Nervous System (CNS) immune landscape and anatomy, which define the “soil” into which circulating tumor cells must seed. Contrary to the long-held notion of an “immune-privileged” site, the brain harbors a specialized and active immune environment (10). The BBB is not merely a physical barrier but a dynamic interface composed of tight junction–coupled brain endothelial cells, basement membrane, pericytes, and astrocytic end-feet. This neurovascular unit strictly regulates the trafficking of molecules and cells and represents the first major obstacle for both metastatic cells and therapeutics (11). The brain parenchyma is patrolled by resident macrophages of distinct origins and locations (12). Microglia, derived from the yolk sac, are the primary innate immune cells of the CNS; they sense disease and injury and can adopt diverse activation states (13). In addition, border-associated macrophages (BAMs) comprise macrophage populations situated in specific anatomical niches (12). Perivascular macrophages reside around blood vessels; they are among the first CNS cells to encounter circulating tumor cells and play a key role in the initial response to metastasis (14). Meningeal macrophages are located within the meninges surrounding the brain. Choroid plexus macrophages reside in the CSF-producing choroid plexus. The specific anatomical site of the metastatic lesion (e.g., parenchyma, leptomeninges) determines which resident cell populations are engaged first, thereby shaping subsequent immune responses and the recruitment of TAMs (14).

## 3 Biological characteristics of TAMs: origin, subtypes, and polarization

TAMs are a core immune component in the TME of brain tumors, playing crucial roles in both brain metastases and primary brain tumors such as glioblastoma (15). The biological characteristics of TAMs include multiple origins, highly heterogeneous subtype classifications, and plastic polarization states, which collectively regulate tumor progression and immune responses (16). This section systematically elucidates the origin, subtypes, and polarization mechanisms of TAMs polarization. Critically, the origin of TAMs is not merely a matter of lineage tracing but is functionally consequential. Their embryonic (microglial) versus adult hematopoietic (monocyte-derived) origin equips them with distinct epigenetic landscapes and functional potentials. This foundational difference means that microglia and monocyte-derived macrophages may respond differently to similar signals within the TME, contributing to a spectrum of phenotypes that the simplistic M1/M2 framework fails to capture. Understanding this origin-dependent heterogeneity is key to developing precise therapeutic strategies.

### 3.1 Origin: resident microglia and peripherally derived macrophages

TAMs are primarily composed of two cell types: resident microglia and peripherally recruited macrophages (17). Microglia originate from myeloid progenitors in the embryonic yolk sac. After migrating to the central nervous system (CNS), they become resident immune cells with self-renewing capabilities and adapt their morphology and functions in response to changes in the microenvironment (18). During tumor progression, peripheral monocytes are recruited by tumor-derived signals such as CCL2 and CSF1. These monocytes infiltrate the TME by crossing the BBB and differentiate into macrophages (19). This recruitment relies on chemokine receptor signaling axes, such as CCR2 and CSF1R (20). In models of GBM and brain metastasis, it has been shown that peripherally derived macrophages can constitute 30-50% of the total TAM population (21). Single-cell RNA sequencing (scRNA-seq) data further reveal that in GBM, there are differences in the transcriptomes and spatial distributions of microglia and peripherally derived macrophages. For example, microglia are more likely to be located at the tumor margins, whereas peripherally derived macrophages are enriched in the tumor core (22). Critically, the origin of TAMs is closely linked to their spatial distribution within the brain tumor microenvironment. As mentioned, scRNA-seq and spatial transcriptomics reveal that microglia-derived TAMs are often enriched at the tumor-brain interface, where they may interact with infiltrating tumor cells and respond to local damage signals. In contrast, peripherally derived macrophages are frequently found in the tumor core and perivascular regions, reflecting their recruitment through the disrupted BBB (17). This spatial partitioning suggests non-redundant roles: microglia might be key in initial tumor cell recognition and containment, while blood-derived macrophages may dominate within established metastases, promoting growth and immunosuppression.

### 3.2 Subtypes: M1 and M2 polarization states and their heterogeneity

TAMs can be classified into classically activated M1 and alternatively activated M2 subtypes based on surface markers, cytokine secretion, and transcriptional profiles. While this dichotomy provides a useful conceptual framework for *in vitro* studies, it represents an oversimplification of the dynamic and plastic states found *in vivo*, particularly within the complex brain TME. In the context of brain metastases, the M1/M2 paradigm is insufficient to explain the observed functional diversity, which is heavily influenced by anatomical location and cellular origin. For instance, scRNA-seq studies reveal that microglia-derived TAMs often exhibit a transcriptomic signature distinct from monocyte-derived TAMs, even when sharing some common M2-like markers. A microglial TAM at the tumor-brain interface may play a role in initial tumor cell recognition and containment, while a monocyte-derived TAM in the hypoxic tumor core might be specialized in promoting angiogenesis and immunosuppression. M1-type TAMs are induced by signals such as IFN-γ, LPS, or GM-CSF, and they express markers including CD80, CD86, MHC-II, IL-12, and TNF-α (23). These M1 TAMs exhibit pro-inflammatory and anti-tumor properties, which include activating Th1 immune responses, enhancing antigen presentation, and directly killing tumor cells (24). In contrast, M2-type TAMs are driven by IL-4, IL-13, IL-10, or TGF-β and express markers such as CD206, ARG1, IL-10, and VEGF (25). M2 TAMs perform immunosuppressive and pro-tumor functions, such as promoting angiogenesis, facilitating tissue repair, and inhibiting T-cell functions (26). In brain tumors, M2-type TAMs typically predominate and are associated with tumor progression, therapeutic resistance, and poor prognosis (27). For example, in brain metastases from breast cancer, M2-type TAMs suppress CD8+ T-cell activity by secreting IL-10 and TGF-β (28). However, it is crucial to note that the M1/M2 paradigm, largely derived from *in vitro* studies, represents a significant oversimplification of the dynamic and plastic states found *in vivo*, particularly within the complex brain TME. The functional diversity of TAMs is better described as a continuum of activation states influenced by a myriad of signals from the microenvironment.

Beyond the M1/M2 Dichotomy, scRNA-seq and Spatial Transcriptomics Reveal Significant Heterogeneity in TAMs. The traditional M1/M2 dichotomy, while useful for conceptualizing macrophage polarization *in vitro*, is increasingly recognized as an oversimplification of the complex and dynamic functional states of TAMs *in vivo*. High-resolution techniques such as scRNA-seq and spatial transcriptomics have unveiled a spectrum of specialized TAM subpopulations that transcend this binary classification, each with distinct transcriptional profiles and roles in brain metastases and GBM (29). 1) immunoregulatory TAMs encompasses several subsets adept at suppressing anti-tumor immunity. A key example is Lipid-Associated Macrophages (LAMs), characterized by high expression of TREM2 and APOE. LAMs are enriched in hypoxic regions and drive immunosuppression through lipid metabolism (29). Other immunoregulatory TAMs highly express checkpoint molecules like PD-L1 and VISTA, directly inhibiting T-cell function. 2) interferon-primed TAMs defined by a strong transcriptional signature of interferon-stimulated genes (ISGs) and the expression of T-cell chemoattractants like CXCL9 and CXCL10. They are thought to represent a potent, potentially anti-tumorigenic axis within the TME, capable of recruiting and activating cytotoxic T cells. 3) pro-angiogenic TAMs promote tumor vascularization by secreting factors such as VEGF, PDGF, and SPP1 (osteopontin). They are often localized to perivascular niches and are critical for supporting vessel co-option and angiogenesis, thereby facilitating tumor growth and metastasis. 4) inflammatory macrophages express markers like CX3CR1 and CCL3, these cells typically exhibit patrolling functions. However, within the brain TME, they can be co-opted to adopt a pro-tumor phenotype (29). Notably, the loss of CX3CR1 expression in microglia—a common feature in brain metastases (BrM)—can promote a supportive niche for BrM growth by recruiting CD68+ myeloid cells that express high levels of immunosuppressive markers like PD-L1 and VISTA (**Figure 1**) (30). 5) transitional Subpopulations co-express markers associated with both M1 (e.g., CD86) and M2 (e.g., CD206) states, reflecting their high functional plasticity and capacity to adapt to dynamic microenvironmental cues (31). This refined heterogeneity is driven by a confluence of tissue-specific signals. For instance, within the brain TME, factors derived from neurons (e.g., BDNF, glutamate) and tumor cells can critically regulate TAM polarization and functional specialization (32).

### 3.3 Polarization mechanisms: signaling pathways and microenvironmental regulation

The polarization of TAMs is intricately regulated by cytokines, metabolites, and cell-cell interactions within the TME. In the STAT signaling pathway, STAT1 activation promotes M1 polarization (e.g., through IFN-γ), whereas STAT3 and STAT6 drive M2 polarization (e.g., through IL-6 and IL-10) (29). In GBM, tumor-secreted IL-11 enhances TRAIL expression in astrocytes via the GP130-STAT3 axis, indirectly influencing TAM function (30–32). Within the NF-κB pathway, pro-inflammatory signals such as TNF-α induce M1 phenotypes by activating NF-κB. However, tumor-associated hypoxia inhibits this pathway via HIF-1α, biasing TAMs towards M2 polarization (33). Metabolic reprogramming also plays a role; lactate accumulation and hypoxic conditions upregulate M2-associated genes such as ARG1 through HIF-1α (34). In addition, HIF-1α activation in TAMs under hypoxia is a key driver of the M2-like phenotype, as highlighted in recent studies (35). Lipid metabolites such as PGE2 promote M2 polarization via EP2/EP4 receptors (36). In terms of cell-cell contact, tumor cells express “don’t eat me” signals such as CD47, which TAM phagocytosis via SIRPα, thereby maintaining an M2 state (37). The polarization status of TAMs is reversible; external interventions such as CSF1R inhibitors (e.g., PLX3397) or TLR agonists can reprogram M2-type TAMs into an M1-like phenotype, enhancing anti-tumor immunity (38). Additionally, microbial elements such as bacterial LPS can modulate TAM polarization via TLR4 signaling, although the role of this pathway in brain tumors remains under investigation (39). In summary, the origin, subtypes, and polarization states of TAMs collectively underpin their biological characteristics, influencing immune evasion and therapeutic responses in brain tumors. Understanding these properties lays the foundation for developing immunotherapeutic strategies targeting TAMs.

## 4 The role of TAMs in brain metastasis: from pre-metastatic niche formation to colonization

TAMs play a critical role in brain metastasis, exerting key regulatory functions from the early formation of the pre-metastatic niche to late-stage tumor colonization. By secreting cytokines, remodeling of the extracellular matrix (ECM), regulating angiogenesis, and immunosuppressing, TAMs facilitate multiple steps in brain metastasis. The diverse functions of TAMs in brain metastasis are not executed by a uniform cell population but are allocated to specialized subpopulations. The biological characteristics of TAMs—particularly their origin and polarization state, as detailed in Section 1—directly determine their functional specialization within the TME. The following sections will dissect the metastatic process by linking specific TAM subsets to their mechanistic roles, thereby illustrating the critical ‘heterogeneity-to-function’ relationship in brain metastasis.

### 4.1 Pre-metastatic niche formation: TAMs drive microenvironment preparation through cytokines and ECM remodeling

The pre-metastatic niche represents the “soil” preparation phase prior to tumor cell colonization, and TAMs facilitate its formation through various mechanisms (**Figure 1**) (40). In the early stages of brain metastasis, factors secreted by the primary tumor, such as exosomes and cytokines, activate resident microglia and recruit peripheral monocytes that differentiate into macrophages, inducing them to polarize towards a pro-tumor phenotype (41). For instance, the tumor exosomal protein CEMIP promotes the colonization of brain metastatic cancer cells (**Figure 1**) (42). In addition, These populations play distinct roles: peripherally derived macrophages, recruited via the CCL2-CCR2 axis, are often the primary source of immunosuppressive cytokines like IL-10 and TGF-β, which potently inhibit anti-tumor immunity (43). In contrast, resident microglia, being the first responders, are crucial for initial ECM remodeling​ through the upregulation of MMPs like MMP9, preparing the ‘soil’ for incoming tumor cells (44). TAMs secrete immunosuppressive cytokines, including IL-4, IL-10, and TGF-β, which inhibit local anti-tumor immune responses by reducing the activity of CD8+ T cells and the cytotoxicity of NK cells, and enhance vascular permeability to facilitate tumor cell infiltration (4). For instance, in a breast cancer brain metastasis model, IL-10 secreted by TAMs suppresses microglial antigen presentation via the STAT3 signaling pathway, thereby reducing immune surveillance (4).

Meanwhile, TAMs promote pre-metastatic niche formation through ECM remodeling. TAMs facilitate the formation of the pre-metastatic niche by remodeling the ECM. They upregulate the expression of matrix metalloproteinases (MMPs), such as MMP2 and MMP9, as well as cathepsins, which degrade basement membrane and ECM components, such as collagen and laminin. This degradation softens the brain tissue matrix, thereby enhancing the invasive capacity of tumor cells (45). scRNA-seq data reveal that in the brain metastatic microenvironment, TAM subpopulations (e.g., M2-like macrophages) highly express ECM-related genes (such as FN1 and SPP1), which are associated with the formation of fibrotic networks (46). Spatial transcriptomics further indicates hat areas of TAM aggregation highly overlap with ECM remodeling hotspots, such as regions of fibrin deposition, confirming their direct involvement in microenvironmental modification (47).

### 4.2 Tumor cell extravasation, vessel co-option, and immune suppression: multifunctional support by TAMs

During the hematogenous metastasis of tumor cells to the brain, TAMs support tumor cell extravasation, vessel co-option, and immune suppression (**Figure 1**). Microglia activated via IL-6 and CSF1 promote the migration of tumor cells across the BBB, thereby facilitating early brain metastasis (48, 49).​ In addition, Microglia have been shown to assist brain invasion in a WNT pathway-dependent manner, an effect that can be reversed by WNT inhibition or LPS-induced activation (50). TAMs secrete chemokines such as CCL2 and CXCL12 to attract circulating tumor cells (CTCs) to the brain vasculature endothelium and express adhesion molecules like ICAM-1 and VCAM-1 to facilitate tumor cell attachment (51). During extravasation, recently recruited monocytes-derived macrophages, due to their proximity to vasculature, are key players in secreting ANGPTL4 and VEGF to disrupt BBB integrity. Meanwhile, activated microglia​ have been shown to form ‘microenvironmental bridges’ that provide physical support for tumor cell extravasation (52–54). In an experimental melanoma model, intravital imaging confirmed that activated TAMs participate in metastasis formation by disrupting BBB integrity via MMP3 (55).​ In a melanoma brain metastasis model, TAMs form “microenvironmental bridges” with endothelial cells, providing direct physical support for tumor cell extravasation (56). Moreover, TAMs promote tumor cells’ utilization of existing vascular networks for growth (vessel co-option) rather than relying on angiogenesis. TAMs secrete pro-angiogenic factors such as VEGF and PDGF to maintain vascular integrity and guide tumor cell migration along the vessel walls through Ephrin signaling (57). In glioblastoma and brain metastases, TAMs are enriched in perivascular regions, co-localizing with tumor cells to form “vessel co-option units” (58). TAMs also create an immunosuppressive microenvironment through multiple mechanisms to inhibit anti-tumor immunity: 1) They express immune checkpoint molecules such as PD-L1 and B7-H3, directly inhibiting T cell function (59). 2) TAMs secrete inhibitory cytokines like TGF-β and IL-10, which induce the expansion of regulatory T cells (Tregs) and myeloid-derived suppressor cells (MDSCs) (45). TAMs secrete chemokines such as CCL3 and CCL4, which are central to the recruitment of lymphocytes; however, this recruitment can be skewed towards immunosuppressive subsets or contribute to an exhausted T cell phenotype (60). Through metabolic reprogramming involving arginase-1 (ARG1) and inducible nitric oxide synthase (iNOS), TAMs deplete essential nutrients like arginine required for T cell function (45). The immunosuppressive landscape is shaped by specific TAM subsets. M2-like, monocyte-derived TAMs are predominant expressers of immune checkpoint molecules like PD-L1. In contrast, lipid-associated macrophages (LAMs), which can originate from both lineages, contribute to immunosuppression through metabolic mechanisms such as arginine depletion via ARG1. Single-cell analysis reveals that in brain metastasis, the spatial interactions between TAMs and T cells lead to upregulation of T cell exhaustion markers such as TIM-3 and LAG-3 (61).

### 4.3 Case studies: mechanisms of TAMs in brain metastases of breast cancer and lung cancer

TAMs play a central role in the progression of BM by modulating the TME to promote tumor cell survival, invasion, and immune evasion (62). This section focuses on the molecular mechanisms of TAMs in brain metastases of breast cancer and lung cancer, including pathway activation, BBB disruption, and remodeling of the immune microenvironment. The anatomical and cellular context of brain metastases significantly influences TAM composition and function, and this varies by primary tumor origin (63).​ Breast cancer brain metastases (BCBM) frequently exhibit a vessel co-option​ growth pattern, where tumor cells grow along pre-existing vessels (64). In this perivascular niche, TAMs (particularly peripherally derived macrophages) interact closely with endothelial cells and astrocytes, driving immune suppression through mechanisms like PD-L1 expression. In contrast, non-small cell lung cancer (NSCLC) brain metastases often form more expansive, solid lesions within the parenchyma (65). Here, the interaction involves both recruited macrophages and resident microglia, with the latter playing a prominent role in ECM remodeling via MMP9 (66). Furthermore, the location itself (e.g., parenchymal vs. leptomeningeal metastasis) dictates the available resident macrophage pool; leptomeningeal disease may involve a greater influence of meningeal macrophages. Therefore, the TAM phenotype and function are not identical across metastases but are shaped by a combination of the primary tumor’s biological instruction and the specific brain micro-anatomy they colonize (12).

#### 4.3.1 Breast cancer brain metastasis: invasion and immune suppression mediated by ANGPTL4 and Src signaling pathways

In BCBM, the TAM population is often dominated by peripherally recruited macrophages, which aligns with the prominent role of the ANGPTL4 pathway​ in disrupting the BBB, a function particularly associated with this subset. ANGPTL4 secreted by TAMs binds to integrin α5β1 on brain endothelial cells, activating the RhoA/ROCK pathway, which leads to endothelial cell contraction and downregulation of tight junction proteins such as occludin. This increases BBB permeability, facilitating tumor cell extravasation (62, 67). In line with this, breast cancer cell-derived ANXA1 guides microglial migration prior to metastatic lesion formation, and ANXA1 induces microglial activation via STAT3 (**Figure 1**) (68).​*In vivo* experiments demonstrate that knocking down ANGPTL4 in TAMs significantly reduces the number of brain metastases from breast cancer (62, 69). Additionally, cytokines secreted by TAMs, such as IL-6, activate Src kinases in tumor cells, phosphorylating downstream targets such as FAK and paxillin, thereby enhancing cell motility and invasiveness (62, 67). Src signaling also promotes tumor cell adhesion to the extracellular matrix (ECM), facilitating the formation of micrometastases (62, 67). Spatial transcriptomics analysis confirms that regions enriched with TAMs in BCBM samples are associated with elevated expression of Src pathway genes such as SRC and PTK2 (FAK), highlighting the role of TAMs in driving tumor progression (62, 63). The TME in BCBM exhibits significant immunosuppressive characteristics, including enrichment of FOXP3+ regulatory T cells (Tregs), CCL18+ M2 macrophages, and LGALS1+ microglia (70, 71). This environment actively inhibits CD8+ T cell activation and promotes immune evasion via PD-1/PD-L1 interactions (71). Studies also indicate that the accumulation of M2 microglia/macrophages in HER2-positive brain metastases is associated with poor prognosis (71).

#### 4.3.2 Lung cancer brain metastasis: MMP9-mediated blood-brain barrier disruption and unique immune landscape

In NSCLC brain metastasis, resident microglia​ play a more prominent role in the initial response. This is consistent with the key mechanism of MMP9-mediated BBB disruption, as microglia are potent producers of MMPs in the CNS (72). Within the TME, TAMs are activated by tumor cell-derived factors such as TGF-β and EGF, and polarize into an M2 phenotype characterized by high MMP9 expression and secretion (66). MMP9 degrades ECM components of the BBB, such as type IV collagen and laminin, leading to loss of barrier function and increased vascular permeability (73). Furthermore, tumor-derived IL-6 facilitates microglial colonization by suppressing their inflammatory phenotype via the JAK2/STAT3 axis in NSCLC brain metastasis. Elevated serum IL-6 levels are associated with the incidence of brain metastasis and patient survival (**Figure 1**) (49).​ Additionally, TAM-derived MMP9 activates latent VEGF, enhancing vascular leakage and further facilitating tumor cell traversal across the BBB (74). Clinical sample analysis shows strong co-localization of TAMs (CD68+ cells) with MMP9 expression, with MMP9 levels positively correlating with metastatic burden (45). Inhibition of TAM activity, such as using CSF1R inhibitors, can reduce MMP9 expression and decrease the incidence of brain metastasis in preclinical models (56). The immune microenvironment of NSCLC brain metastases differs from that of the primary tumor, exhibiting a more pronounced immunosuppressive profile. Compared to primary lung tumors, brain metastases display a lower proportion of tumor-infiltrating lymphocytes (TILs), reduced PD-L1 expression, and upregulation of anti-inflammatory markers such as TOLLIP and HLA-G (65, 75, 76). Notably, in EGFR-mutant brain metastases, immune-related pathways are upregulated, whereas TP53 mutations are associated with increased CD8+ T cell infiltration and aggregation of immunosuppressive myeloid cells (65). This heterogeneity in the immune landscape underscores the interaction between the TME and tumor genotype.

#### 4.3.3 Melanoma brain metastases: A unique immune microenvironment and responses to targeted and immunotherapy

Melanoma has a striking propensity for brain metastasis; autopsy series have shown that up to 50% of patients with advanced melanoma develop brain metastases (77). The tumor microenvironment of melanoma brain metastases (MBM) exhibits features distinct from other cancer types, which profoundly influence clinical behavior and therapeutic response (78). First, BRAF mutations occur in approximately 50% of melanomas, and aberrant activation of the downstream MAPK signaling pathway not only drives tumor growth but also actively shapes the immune microenvironment (79). BRAF mutations may promote a TAM phenotype with both inflammatory and immunosuppressive characteristics by secreting specific factors such as VEGF, IL-6, and IL-10 (79). Targeted therapy with BRAF inhibitors (e.g., dabrafenib) and MEK inhibitors (e.g., trametinib) can directly suppress tumor cell proliferation and indirectly affect TAMs, reprogramming them toward a proinflammatory state and thereby enhancing T-cell infiltration and function (80). Second, interactions between melanoma cells and TAMs/microglia are particularly pronounced. MBM often displays prominent lymphocytic infiltration, which coexists with a strong immunosuppressive state. Single-cell sequencing studies reveal that TAMs in MBM highly express multiple immune checkpoint molecules, such as PD-L1, LAG-3, and VISTA, providing the molecular basis for responses to immune checkpoint inhibitors but also contributing to resistance (81, 82). Studies suggest that TAMs in MBM recruit lymphocytes through axes such as CXCL10–CXCR3, while simultaneously inducing T-cell exhaustion via the aforementioned checkpoint molecules, creating a “recruit-and-suppress” paradox (83). Third, the growth pattern of melanoma brain metastases also influences the distribution and function of TAMs. Melanoma cells tend to grow along pre-existing vessels (vascular co-option). This perivascular distribution brings tumor cells into close contact with resident microglia, which support tumor survival by secreting trophic factors such as GDNF (78).

In summary, melanoma brain metastases represent a unique model characterized by intense TAM involvement and intertwined immunosuppressive and inflammatory signaling. Understanding this distinctive microenvironment is critical for optimizing therapeutic strategies. Combining BRAF/MEK-targeted therapy with TAM-reprogramming strategies (e.g., CSF1R inhibitors) or immune checkpoint inhibitors is emerging as a promising approach to overcome therapeutic resistance (8, 9) (see related clinical trials in **Table 1**).

**Table 1:** Clinical trials directed towards brain metastases in recent years.

BM type	Identifier	Treatment	Patients included	Phase	Status
SCLC	NCT03297788	SRS or WBRT	SCLC patients with BM	II	Completed
NSCLC	NCT04824079	Keynatinib	Advanced NSCLC patients with BM after treatment with EGFR inhibitors	II	Recruiting
NSCLC	NCT05012254	Treatment with two cycles of Platinum-based chemotherapy (Carboplatin or Cisplatin) plus Nivolumab and Ipilimumab; then maintain with Nivolumab and Ipilimumab	Stage IV or recurrent, NSCLC patients with synchronous BM	II	Active, not recruiting
SCLC	NCT04631029	Entinostat in combination with Atezolizumab/Carboplatin/Etoposide	Extensive stage SCLC patients with BM	I	Completed
NSCLC	NCT05104281	Osimertinib combined with Bev-acizumab	NSCLC patients with BM and EGFR mutation	I	Recruiting
NSCLC	NCT06128148	JYPO322 (an orally available inhibitor of ROS1)	NSCLC patients with BM and ROSI fusion	I	Recruiting
NSCLC	NCT04967417	and Pembrolizumab or Pacli-taxel, Carboplatin and Pembroli-zumab	Stage IV NSCLC patients with BM Pemetrexed, Carboplatin	II	Recruiting
NSCLC	NCT05948813	TY-9591; Osimertinib	Patients with EGFR-mutated	II	Recruiting
NSCLC	NCT06501391	PD-L1/PD-1 inhibitor and chemotherapy combined with SRT or WBRT	Stage IV NSCLC patients with BM and without driver gene muta-	II	Recruiting
NSCLC	NCT06676917	Datopotamab Deruxtecan	Non-squamous NSCLC with BM	II	Not yet recruiting
BC	NCT03696030	Autologous HER2-targeted chimeric antigen receptor	HER2 positive BC patients with BM	I	Recruiting
BC	NCT05781633	A regimen of eutidrone, etopo-side and bevacizumab	BC patients with BM	II	Recruiting
BC	NCT06152822	Pyrotinib combined	HER2 positive BC patients with BM	II	Recruiting
BC	NCT05872347	SPH4336 (a novel highly selective oral CDK4/6 inhibitor)	HR-positive, HER2-negative BC patients with BM	II	Recruiting
BC	NCT06088056	SRT combined with Trastu-zumab-Deruxtecan (T-DXd)	HER2 positive BC patients with BM	II	Not yet recruiting
BC	NCT06418594	Adebrelimab plus apatinib and etoposide	HER2-negative BC patients with BM	II	Recruiting
BC	NCT06462079	Sacituzumab Govitecan	HER2-negative BC patients with BM	II	Not yet recruiting
BC	NCT06210438	SHR-A1921 (an antibody conjugated drug targeting Trop-2) combined with bevacizumab	Triple-negative BC patients with BM	I	
Melanoma	NCT03898908	Encorafenib and binimetinib	Patients with BRAF mutant melanoma metastatic to the brain	II	Active, not recruiting
Melanoma	NCT04074096	Adding upfront SRS to bini-metinib-encorafenib-pembroli-	Melanoma BRAFV600 mutation-positive melanoma patients with BM	I	Active, not recruiting
Melanoma	NCT05704933	zumab combination therapy	Melanoma Melanoma patients with BM	I	Active, not recruiting

BC, breast cancer; CRT, cranial radiation therapy; EGFR, Epidermal Growth Factor Receptor; SRT, stereotactic radiotherapy; SRS, stereotactic radiotherapy; WBRT, whole brain radiation therapy.

## 5 Mechanistic in-depth analysis: key signaling pathways in TAM and tumor cell interactions

The 6interactions between TAMs and brain metastatic cells involve complex signaling pathways that coordinate tumor progression, immune evasion, and treatment resistance. This section provides a detailed analysis of the key mechanisms underlying TAM and tumor cell interactions, including neuro-cancer interactions, immunometabolic regulation, and intercellular communication. The focus will be on elucidating the molecular basis and functional impacts of these signaling pathways.

### 5.1 Neuro-cancer interactions: neuronal activity drives brain metastasis via soluble factors and synaptic signaling

The interaction between neurons and tumor cells is a unique characteristic of the brain metastatic microenvironment, with TAMs playing a mediating role. Neuronal activity promotes tumor growth and invasion either directly or indirectly through TAMs by secreting neurotrophic factors and neurotransmitters. Neuron-derived brain-derived neurotrophic factor (BDNF) activates the MAPK and PI3K/AKT pathways in tumor cells via its receptor TrkB, enhancing tumor cell survival, proliferation, and migration (84). In models of breast cancer brain metastasis, the BDNF/TrkB axis upregulates MMP9 expression, which facilitates BBB disruption and tumor cell extravasation (85). Additionally, glutamate released from neuronal activity activates calcium signaling pathways within tumor cells through NMDAR (N-methyl-D-aspartate receptor), driving cell cycle progression and invasion (86). In GBM, NMDAR signaling enhances synapse-like connections between tumor cells and neurons, promoting network integration (87). TAMs modulate NMDAR expression by secreting IL-6 and TGF-β, indirectly influencing tumor-neuron interactions (88, 89). Furthermore, thrombospondin-1 (TSP1), secreted by neurons and TAMs, promotes synaptogenesis by binding to calcium channel subunits (e.g., α2δ-1) on neurons, enhancing tumor cell colonization in the brain (90). Additionally, microglial subsets expressing CD74 contribute to metastatic growth by secreting midkine (MDK), a pleiotropic factor that promotes tumor cell survival and invasion (**Figure 1**) (91). In GBM models, high TSP1 expression correlates with synapse density at the invasive tumor front, and TSP1 secretion by TAMs is regulated by STAT3 signaling (92, 93). Spatial transcriptomics reveal that TSP1+ TAMs aggregate at the tumor-brain interface and colocalize with neuronal synaptic markers like SYN1 (93). Neuro-cancer interactions also involve electrical signal coupling. Tumor cells mimic neuronal activity by expressing voltage-gated ion channels such as Nav1.5, recruiting TAMs to perisynaptic regions (94). This interaction creates “neuro-tumor synapses,” facilitating the integration of tumor cells into the brain’s neural network and evading immune surveillance.

Beyond neurotrophic factors, classical neurotransmitters released by active neurons serve as direct signals for TAMs, which express a repertoire of neurotransmitter receptors. The recognition of these signals significantly influences TAM polarization and function. Glutamate, the primary excitatory neurotransmitter, can signal to TAMs through both ionotropic (e.g., N-methyl-D-aspartate receptors, NMDARs) and metabotropic glutamate receptors (mGluRs) (95). Excessive glutamate release, a common feature in the perturbed brain TME, leads to sustained activation of these receptors on TAMs. Signaling through the GluN2B subunit of NMDARs can activate the PI3K/AKT and MAPK pathways, promoting a transcriptional program that enhances the expression of pro-tumorigenic factors such as VEGF and IL-10, thereby reinforcing an M2-like, immunosuppressive phenotype (96, 97). Conversely, activation of certain mGluRs (e.g., mGluR2/3) has been linked to the suppression of NF-κB signaling, potentially dampening beneficial anti-tumor inflammatory responses in TAMs (98). GABA (γ-aminobutyric acid), the main inhibitory neurotransmitter, also plays a crucial role. TAMs express functional GABA-A receptors (ligand-gated chloride channels) and GABA-B receptors (G-protein coupled receptors). GABAergic signaling generally exerts an immunosuppressive effect on myeloid cells. Activation of GABA-A receptors leads to an influx of chloride ions, causing membrane hyperpolarization which can inhibit the production of pro-inflammatory cytokines like IL-1β and TNF-α (99). Signaling through GABA-B receptors can inhibit adenylate cyclase, reducing intracellular cAMP levels and modulating downstream pathways that ultimately limit TAM activation and promote an M2-like state (100). This GABA-mediated suppression may contribute to the immune-privileged nature of the brain metastatic niche by restraining TAMs’ anti-tumor capabilities.

The crosstalk between neurotransmitter systems and classical immune signaling pathways (e.g., cytokine receptors) creates a complex regulatory network within TAMs. For instance, glutamate signaling can synergize with IL-10/STAT3 signaling to enhance immunosuppressive gene expression (101). This intricate interplay positions neurotransmitters as critical microenvironmental cues that skew TAMs toward pro-metastatic functions, including support for tumor cell survival, angiogenesis, and immune evasion. Targeting these neurotransmitter receptors on TAMs (e.g., with receptor antagonists) represents a novel therapeutic avenue to disrupt the neuro-cancer axis and revert TAM-mediated immunosuppression in brain metastases.

### 5.2 Immunometabolic regulation: lipid metabolism and hypoxia response in TAMs drive immune suppression

TAMs adapt to the unique brain TME through metabolic reprogramming, with their lipid metabolism and hypoxia response pathways directly regulating immunosuppressive functions. Upregulation of lipid metabolism genes (e.g., SPP1, APOE, and LPL) in TAMs promotes cholesterol and fatty acid accumulation, driving M2 polarization (59). In GBM and brain metastasis, SPP1+ TAM subsets enhance invasiveness by secreting osteopontin, which activates CD44 and integrin signaling within tumor cells (4, 102). Lipid metabolites (such as prostaglandin E2 and sphingosine-1-phosphate) suppress T-cell activity and NK cell cytotoxicity through G-protein coupled receptors (e.g., EP2 and S1PR1) (103). scRNA-seq analysis reveals that lipid-associated TAMs (LAMs) are enriched in hypoxic regions and positively correlate with the expression of immune checkpoint molecules (such as PD-L1) (82). It is important to note that while many of these immunometabolic mechanisms (e.g (39, 90, 92).,) have been characterized in peripheral or general tumor models, emerging evidence supports their critical role in shaping the immunosuppressive landscape of brain metastases. In the HIF (hypoxia-inducible factor) pathway, the hypoxic brain TME stabilizes HIF-1α and HIF-2α, inducing TAMs to express VEGF, ARG1, and IL-10, which promote angiogenesis and immune suppression (104). HIF signaling also upregulates glycolytic enzymes (e.g., HK2 and LDHA) in TAMs, acidifying the microenvironment through lactate accumulation and further inhibiting T-cell function (105). In lung cancer brain metastasis, HIF-1α-activated TAMs recruit MDSCs through the CXCL12/CXCR4 axis, amplifying the immunosuppressive network (4). TAMs compete with tumor cells for nutrients (such as glucose and glutamine) and coordinate metabolic adaptation via mTORC1 signaling (59). For instance, exosomes derived from TAMs carry metabolic enzymes (such as ENO1 and GAPDH), enhancing tumor cell glycolysis and supporting their proliferation (59). Metabolite exchange (such as lactate and ketone bodies) also regulates immune gene expression through epigenetic modifications (such as histone acetylation) (106). Additionally, disulfide stress is an emerging concept in the TME that regulates the function of TAMs. This stress state is triggered by the intracellular accumulation of disulfides (such as cystine) and is closely linked to glutamine metabolism and antioxidant responses (107). Under cystine-rich conditions in the TME, TAMs take up large amounts of cystine via the cystine/glutamate antiporter xCT, leading to intracellular disulfide stress. This not only promotes M2-like polarization and immunosuppressive functions but may also influence signaling pathway activity by altering protein disulfide-bond modifications. Targeting disulfide stress–related pathways (e.g., inhibiting xCT) has been shown to remodel the TME and reprogram TAMs toward an antitumor phenotype, representing a new therapeutic avenue for metabolic regulation of TAMs (108).

### 5.3 Intercellular communication: exosome and cytokine networks coordinate TAMs functions

The communication between TAMs and tumor cells relies on exosome-mediated molecular transfer and autocrine/paracrine cytokine signaling networks, which finely regulate tumor progression. Tumor cell-derived exosomes carry non-coding RNAs (such as miR-21 and lncRNA H19), proteins (such as PD-L1 and MMP2), and metabolites, which are endocytosed by TAMs and modulate their polarization (109–112). In breast cancer brain metastasis, exosomal miR-21 targets PTEN to activate the PI3K/AKT pathway in TAMs, promoting IL-10 secretion (113, 114) (**Figure 1**). Conversely, TAM-derived exosomes transfer TGF-β and IL-6 to tumor cells, enhancing epithelial-mesenchymal transition (EMT) and stemness properties (115) (**Figure 1**). Exosomes also mediate communication across the BBB: brain endothelial cell-derived exosomes carrying ICAM-1 promote TAM recruitment to metastatic sites (116). Simultaneously, IL-6 and IL-11 activate the STAT3 signaling pathway via the GP130 receptor, driving TAM M2 polarization and immunosuppressive functions (117). In GBM, tumor-secreted IL-11 induces TAMs to express TRAIL via the IL11RA/STAT3 axis, leading to T-cell apoptosis (118). Single-cell analysis reveals that IL-6+ TAM subsets colocalize with T-cell exhaustion markers (such as TIM-3), and STAT3 inhibitors can reverse this effect (119). Additionally, TAM-derived IL-10 enhances tumor cell IL-6 secretion via STAT3, forming a pro-tumoral loop (45). Subsequently, TAMs directly interact with tumor cells or T-cells through membrane-bound ligands (such as FasL and PD-L1) (45). For instance, TAM-expressed PD-L1 binds to PD-1 on T-cells, inhibiting TCR signaling and cytokine production (120). Moreover, TAMs express integrins (such as αVβ3) that bind to tumor cell ECM proteins (such as fibronectin), activating the FAK/Src pathway and promoting invasion (121).

### 5.4 TAM-mediated resistance to chemotherapy and immunotherapy

Beyond their roles in promoting tumor progression, TAMs are key drivers of resistance to both chemotherapy and immunotherapy, representing a major clinical hurdle. The mechanisms underlying TAM-mediated therapeutic resistance are multifaceted and involve creating a protective niche around tumor cells.

#### 5.4.1 Resistance to chemotherapy

TAMs contribute to chemoresistance through several pathways. TAMs can secrete excessive extracellular matrix (ECM) components, such as collagen and fibronectin, creating a dense physical barrier that impedes the penetration of chemotherapeutic agents into the tumor core. Moreover, TAM-derived factors like prostaglandin E2 (PGE2) can further compromise drug delivery by promoting vascular abnormality. TAMs secrete a plethora of survival factors that directly counteract the pro-apoptotic effects of chemotherapy. For instance, TAM-derived IL-6 and IL-8 activate the STAT3 and NF-κB pathways in tumor cells, upregulating anti-apoptotic proteins (e.g., Bcl-2, Bcl-xL) and enabling tumor cells to survive cytotoxic stress (122). Certain TAM subsets, particularly those with an M2-like phenotype, express high levels of metabolic enzymes like cytidine deaminase, which can directly metabolize and inactivate nucleoside analog drugs such as gemcitabine, thereby protecting neighboring tumor cells (123).

#### 5.4.2 Resistance to immunotherapy

The immunosuppressive functions of TAMs are principal mechanisms of resistance to immune checkpoint inhibitors (ICIs). TAMs are a major non-tumor source of immune checkpoint molecules like PD-L1, PD-L2, B7-H4, and VISTA within the TME. By engaging receptors on T cells, these molecules directly induce T cell exhaustion or anergy, effectively neutralizing the reactivation of anti-tumor immunity intended by ICIs (124). TAM-derived chemokines (e.g., CCL2, CCL22) recruit regulatory T cells (Tregs) and other immunosuppressive myeloid cells, amplifying the immunosuppressive network. TAMs also secrete cytokines like IL-10 and TGF-β, which not only inhibit effector T cell function but also promote the differentiation of naive T cells into Tregs, creating a sustained resistant environment (125). TAMs express high levels of enzymes like indoleamine 2,3-dioxygenase (IDO) and arginase-1 (ARG1). IDO catabolizes tryptophan into kynurenine, which is toxic to T cells and promotes Treg differentiation, while ARG1 depletes arginine, an essential nutrient for T cell function and proliferation, leading to T cell dysfunction (126). (4) Support of Tumor-Initiating Cells (TICs):​ TAMs secrete factors such as IL-6 and TGF-β that help maintain a stem-like, therapy-resistant TIC population. These TICs are often inherently resistant to chemotherapy and can repopulate the tumor after treatment, with their survival being bolstered by interactions with TAMs (127).

## 6 Therapeutic Strategies Targeting TAMs: 9L

**Figure 2:**
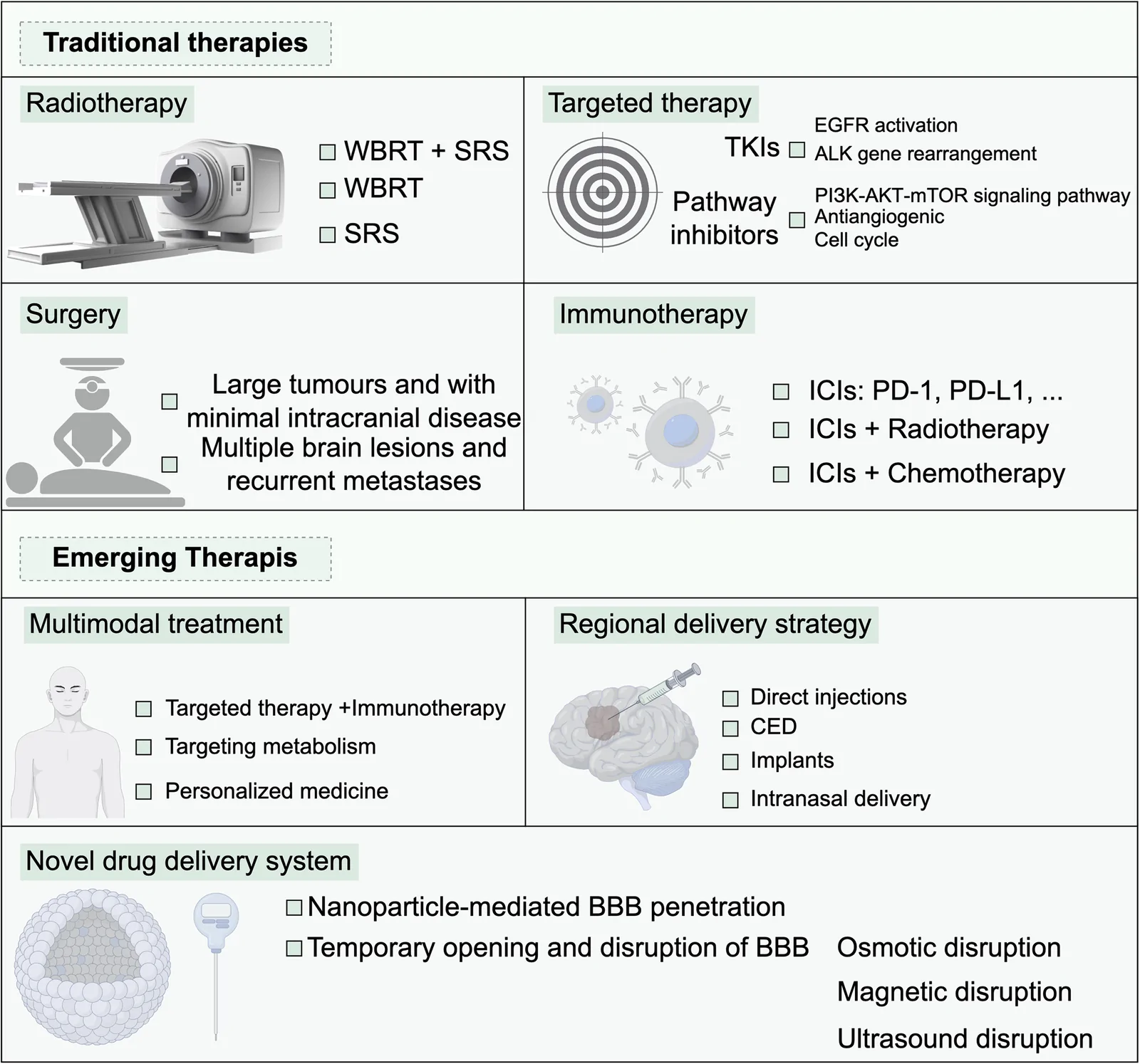
The current treatment strategies for brain metastases include traditional therapies and emerging therapies.

TAMs play a crucial role in brain metastases and primary brain tumors, such as glioblastoma, making them a promising therapeutic target. Strategies targeting TAMs encompass reprogramming their polarization state, combination immunotherapies, nanotechnology, and cell-based therapies (**Figure 2**). These approaches have transitioned from preclinical research to clinical translation. This section systematically elucidates the mechanisms, advancements, and challenges of these therapeutic strategies.

### 6.1 Conventional treatments, emerging therapeutic strategies, and key enabling technologies for brain metastases

This section outlines the therapeutic landscape for brain metastases, covering conventional standard regimens, cutting-edge emerging strategies, and enabling technologies that potentiate treatment efficacy. It clarifies the indications, targets, and combination logic of different modalities, and highlights the shift from single-modality management toward multimodal, precise, and targeted approaches. Traditionally, therapeutic targeting of TAMs has focused on depleting their overall numbers or reprogramming their polarization state from a pro-tumorigenic (M2-like) to an anti-tumorigenic (M1-like) phenotype. However, in line with the profound functional heterogeneity of TAMs, a new paradigm is emerging: the precise targeting of specific TAM subpopulations based on their distinct pro-tumor functions, such as promoting angiogenesis or driving immunosuppression. The following sections will summarize strategies encompassing both conventional reprogramming approaches and these novel subset-specific interventions.

#### 6.1.1 Conventional therapies

Conventional treatment focuses on local control and removal of tumor tissue, with individualized planning based on tumor burden and characteristics of metastatic lesions. The main categories include radiotherapy, surgery, and targeted therapy. Radiotherapy, comprising whole-brain radiotherapy (WBRT) and stereotactic radiosurgery (SRS), is a cornerstone of traditional management for brain metastases. SRS is suitable for relatively small intracranial disease with a limited number of lesions (e.g., minimal intracranial disease), allowing precise targeting of tumor tissue while sparing normal brain. WBRT is indicated for multiple brain lesions, recurrent metastases, or more extensive intracranial disease, and can be combined with SRS (WBRT + SRS) to further improve local control. Surgery is primarily considered for large tumors to debulk disease, alleviate mass effect, and create conditions favorable for subsequent adjuvant therapies. Targeted therapy refers to established regimens directed at specific oncogenic drivers or signaling pathways and is widely used clinically (**Figure 2**).

#### 6.1.2 Emerging therapies

Emerging strategies emphasize precision, combination, and multimodality to overcome the limitations of conventional care, centering on combination regimens, innovations in precision/targeted approaches, and personalized medicine. In combination therapies, targeted therapy plus immunotherapy leverages the precise tumoricidal effects of targeted agents together with the durable anti-tumor immunity of immunotherapy for synergistic benefit. Immune checkpoint inhibitor (ICI)–based combinations commonly include ICIs (e.g., PD-1/PD-L1 inhibitors) with radiotherapy (ICIs + radiotherapy) or chemotherapy (ICIs + chemotherapy), where radiotherapy/chemotherapy provide immunosensitizing effects that enhance ICI-mediated activation of anti-tumor immunity. Multimodal treatment integrates the strengths of multiple modalities to craft individualized combinations tailored to complex clinical scenarios, thereby improving response rates. In precision and targeted innovation, metabolism-directed therapies exploit tumor-specific metabolic reprogramming by targeting key metabolic pathways to restrict energy supply and proliferation. In personalized medicine, treatment is tailored to each patient’s genomic features, immune landscape, and other individual factors to achieve truly bespoke, precision therapy (**Figure 2**).

#### 6.1.3 Key enabling technologies: blood–brain barrier penetration and regional delivery strategies

The BBB is a major bottleneck limiting the efficacy of therapeutics in the brain. Current research highlights two core technological approaches: novel drug delivery systems and regional delivery strategies. Novel drug delivery systems include nanoparticle-mediated BBB penetration and temporary opening or disruption of the BBB. Nanoparticle-based systems harness nanoscale size and targeting ligands to overcome physiological barriers and achieve efficient delivery of therapeutics to intracranial tumor tissue. Temporary BBB opening/disruption methods use physical techniques such as osmotic, magnetic, or ultrasound-mediated disruption to transiently increase permeability and create entry routes for drugs. Regional delivery strategies increase drug concentrations in the brain via local administration, including convection-enhanced delivery (CED), direct injections, implant-based delivery, and intranasal delivery. These approaches reduce systemic toxicities while increasing local tumor drug exposure, thereby enhancing therapeutic effect. In sum, this section presents the evolution and technological advances in the treatment of brain metastases, summarizing the clinical contexts and mechanisms of conventional therapies while highlighting the value of emerging combinations, precision approaches, and innovative delivery technologies, thereby providing a systematic framework to guide clinical decision-making and the development of new therapeutic technologies (**Figure 2**).

### 6.2 Reprogramming TAMs: converting M2 to M1 phenotype

Reprogramming TAMs from a pro-tumoral M2 phenotype to an anti-tumoral M1 phenotype is a core strategy for treating brain tumors. Various small-molecule inhibitors and agonists achieve this by modulating key signaling pathways. The CSF1/CSF1R axis is a primary driver of TAM recruitment and survival (128). CSF1R inhibitors, such as PLX3397 and BLZ945, block this pathway, reducing TAM numbers and promoting M1-like phenotypic conversion (129). In GBM models, PLX3397 treatment decreases TAM infiltration, enhances CD8+ T cell activity, and prolongs survival (128). Preclinical studies show that CSF1R inhibition synergizes with radiotherapy to enhance anti-tumor immunity by reducing immunosuppressive TAMs (130). However, the clinical translation of CSF1R inhibitors has revealed significant limitations. While effective in preclinical models, monotherapy trials (e.g., Phase I/II of PLX3397 in recurrent GBM) often show only modest reduction in tumor growth and limited survival benefit. This limited efficacy is attributed to several factors: (1) Compensatory mechanisms: The depletion of TAMs can be countered by the rapid recruitment of alternative immunosuppressive myeloid cells, such as neutrophils or MDSCs, which fill the vacant niche and maintain immunosuppression. (2) Lack of targeting specificity: CSF1R inhibition affects both pro-tumoral M2-TAMs and potentially beneficial M1-like subsets, potentially blunting the anti-tumor immune response. (3) Tumor heterogeneity: The dependency on CSF1R signaling varies across cancer types and even within different regions of a tumor, affecting response. These challenges underscore that CSF1R inhibition is unlikely to be effective as a standalone treatment and must be strategically combined with other modalities. Toll-like receptor (TLR) agonists, such as TLR7/8 agonist R848 and TLR9 agonist CpG, activate the MyD88/NF-κB pathway, inducing TAM polarization towards the M1 phenotype (131). In brain metastasis models, intracranial injection of R848 increases IL-12 and TNF-α secretion by TAMs, promoting a Th1 immune response and inhibiting tumor growth (132, 133). TLR agonists also enhance antigen presentation and synergize with checkpoint inhibitors. Histone deacetylase (HDAC) inhibitors, such as vorinostat, alter TAM gene expression through epigenetic regulation, decreasing M2-associated genes like ARG1 and IL-10 while upregulating M1 markers such as iNOS and CD86 (134, 135). In GBM, HDAC inhibition reverses the immunosuppressive function of TAMs, enhancing T cell-mediated tumor killing (134). Clinical trials have shown preliminary efficacy of HDAC inhibitors combined with chemotherapy (136–139). Other reprogramming strategies include: 1) Inhibiting the STAT3 signaling pathway, which drives M2 polarization. Inhibitors like WP1066 block the IL-10/STAT3 axis, promoting the conversion of TAMs to the M1 phenotype (140). 2) Targeting TAM metabolism, such as with IDO inhibitors or ARG1 inhibitors, to reverse immunosuppression. For example, the IDO inhibitor epacadostat reduces kynurenine accumulation, enhancing T cell function (141). Reprogramming strategies face challenges such as off-target effects and toxicity, but combination therapies can improve specificity.

While the abundance of pro-tumoral TAMs makes them an attractive target, therapeutic strategies can be broadly categorized into two paradigms: those aiming to deplete TAMs (e.g., CSF1R inhibitors) and those aiming to reprogram or modulate their function (e.g., TLR agonists, HDAC inhibitors). Each approach has distinct advantages and drawbacks. Depleting strategies may rapidly reduce immunosuppressive pressure but can trigger compensatory recruitment mechanisms and eliminate potential anti-tumor functions. Conversely, reprogramming strategies seek to harness the plasticity of TAMs but face challenges in achieving complete and sustained phenotypic conversion within the complex TME. The following sections will critically evaluate these strategies, with a focus on their mechanisms, clinical translation challenges, and comparative potential.

### 6.3 Combination immunotherapy: synergistically enhancing anti-tumor immunity

Monotherapies often face limitations due to tumor heterogeneity and immune evasion mechanisms. Combination strategies, by targeting multiple pathways, synergistically enhance therapeutic efficacy. PD-1/PD-L1 inhibitors (such as nivolumab) release T cell inhibition, but are more effective when combined with TAM-targeted therapies (142). For instance, the combination of anti-CSF1R antibody and anti-PD-1 in GBM models reduces PD-L1 expression on TAMs, enhances T cell infiltration, and boosts T cell function (143, 144). Clinical data indicate that combination therapy improves objective response rates, though managing immune-related adverse events (irAEs) is necessary (143, 144). Oncolytic viruses, such as HSV-1-derived talimogene laherparepvec, are engineered to express therapeutic molecules. These viruses can express TRAIL-blocking single-chain antibodies (scFv) to inhibit TRAIL+ astrocytes-induced T cell apoptosis, thereby enhancing anti-tumor immunity. In GBM models, this therapy prolongs survival and increases tumor-specific T cells (118). Viral replication also directly lyses tumor cells, releasing antigens that promote immune responses (118). Additionally, temozolomide (TMZ), an oral alkylating chemotherapeutic agent and the standard-of-care for glioblastoma due to its ability to cross the blood-brain barrier,​ when combined with anti-PD-1, indirectly affects TAMs by reducing Tregs and MDSCs, though optimizing the timing is crucial to avoid immunosuppression (145, 146). Moreover, combining IL-12 or IFN-γ with TAM-targeted therapies promotes M1 polarization, although systemic toxicity limits their application (147). Combination strategies must be individualized, with therapy choices based on the tumor’s immune phenotype, such as TAM density and PD-L1 expression.

### 6.4 Nanotechnology and cellular therapy: precision delivery and engineered cells for overcoming the BBB

Nanotechnology offers revolutionary approaches to precisely target TAMs by improving drug delivery across the most significant obstacle in neuro-oncology: the BBB. The strategies below focus on leveraging nanoscale properties to achieve intracranial targeting.

#### 6.4.1 Nanoparticle strategies for BBB penetration

Nanoparticles (NPs) can be engineered with specific characteristics to facilitate BBB crossing through several key mechanisms. NPs can be surface-functionalized with ligands that bind to receptors highly expressed on brain endothelial cells, such as transferrin receptor (TfR), low-density lipoprotein receptor (LPR), or insulin receptor. Upon binding, these receptors initiate vesicular transport processes that carry the NP cargo across the endothelial cell layer and into the brain parenchyma. For example, TfR-targeted NPs have demonstrated significantly enhanced accumulation in brain metastases in preclinical models (148). Some NPs are designed to be taken up by circulating immune cells (e.g., monocytes, neutrophils) that possess inherent chemotactic properties to migrate towards inflammatory sites like tumors. These cells act as “Trojan horses,” carrying the therapeutic NPs across the BBB. The previously mentioned MDC-735 system leverages the tumor-homing ability of macrophages for this purpose (149). NPs can be combined with techniques that temporarily increase BBB permeability. This includes co-administration with agents like focused ultrasound (FUS) in conjunction with microbubbles, which non-invasively and locally disrupt tight junctions, creating a temporary window for enhanced NP extravasation (150). The intrinsic properties of NPs—such as size (optimized to be below 100 nm for optimal vascular extravasation), surface charge (neutral or slightly negative to reduce non-specific binding), and surface coating with polyethylene glycol (PEG) to prolong circulation time—are critical for passive targeting and successful BBB traversal (151).

#### 6.4.2 Applications in TAM targeting

Nanotechnology and cellular therapy offer precise targeting methods for TAMs, improving drug delivery and specificity. Nanoparticles loaded with drugs can target TAMs. For example, the MDC-735 (Macrophage-Drug Conjugate) system leverages the tumor-homing ability of macrophages to deliver a potent chemotherapeutic agent. This platform consists of macrophages loaded with ferritin-drug conjugates (HFt-vcMMAE), where human ferritin (HFt) nanocages serve as a carrier for monomethyl auristatin E (MMAE)—a highly cytotoxic microtubule inhibitor. The MMAE is attached to HFt via a protease-cleavable linker (vc), ensuring its release specifically within the tumor microenvironment upon cleavage by cathepsin B, thereby inducing tumor cell apoptosis with reduced off-target effects (149). MDC-735 leverages the tumor-homing ability of macrophages to deliver drugs to the brain TME, subsequently releasing MMAE to induce tumor cell apoptosis (149). Preclinical studies have shown that MDC-735 reduces GL261 glioma growth and enhances T cell responses (149). Engineered macrophages expressing chimeric antigen receptors (CARs) can target tumor antigens. CAR-Ms targeting GBM antigens (such as EGFRvIII) kill tumor cells via phagocytosis and secrete pro-inflammatory cytokines (such as IL-12) (152). In xenograft models, CAR-Ms infiltrate tumors, inhibit growth, and are less susceptible to suppression by the TME (152). CAR-Ms can also synergize with CAR-T cells to enhance antigen presentation (152). Research has demonstrated that drug delivery to the brain TME can be improved under the guidance of external magnetic fields, increasing the specificity of TAM targeting (153). Challenges in cellular therapy include manufacturing complexity, immunogenicity, and *in vivo* persistence, but gene editing technologies such as CRISPR enhance safety.

### 6.5 Targeting functional TAM subpopulations

Moving beyond the M1/M2 paradigm, recent efforts have focused on neutralizing the specific pro-tumor functions of TAM subsets, a strategy that may offer greater precision and reduced off-target effects. Pro-angiogenic TAMs is a key subpopulation of TAMs promotes tumor vascularization through the robust secretion of factors like VEGF, SPP1 (osteopontin), and PDGF. The efficacy of anti-VEGF therapies (e.g., bevacizumab) in brain tumors may partly rely on disrupting the pro-angiogenic function of TAMs. Combining VEGF blockade with TAM-depleting agents (e.g., CSF1R inhibitors) or reprogramming agents has shown synergistic effects in preclinical models by simultaneously targeting both tumor endothelial cells and angiogenic TAMs (154). Directly targeting signaling pathways within TAMs that drive angiogenesis is an emerging approach. For instance, inhibiting the SPP1-CD44 axis or the Angiopoietin-2 (Ang-2) pathway can specifically blunt the pro-angiogenic output of TAMs, normalizing the tumor vasculature and improving drug delivery (155). Instead of broadly depleting all TAMs, strategies aim to neutralize their immunosuppressive mechanisms. Immunosuppressive TAMs, particularly Lipid-Associated Macrophages (LAMs), rely on specific metabolic pathways. Inhibiting the cystine/glutamate antiporter (xCT) can disrupt disulfide stress and impair the immunosuppressive function of LAMs. Similarly, targeting the ARG1 enzyme can reverse arginine depletion and restore T cell function in the TME (108). Antibodies targeting immune checkpoints highly expressed on TAMs, such as anti-LILRB4 or anti-SIRPα (to disrupt the CD47-”don’t eat me” signal), are under clinical investigation. These agents directly enhance the phagocytic activity of TAMs against tumor cells without requiring a full phenotypic switch (156). The success of these subset-specific strategies hinges on accurately identifying the dominant functional TAM population within a patient’s tumor, underscoring the importance of companion biomarkers.

### 6.6 Clinical trial summary: from early to late-stage studies

Therapies targeting TAMs have been evaluated in multiple clinical trials (157). A Phase I/II trial of PLX3397 in recurrent GBM showed reduced TAM infiltration, but limited efficacy as a monotherapy (median PFS of 2.3 months); however, combining it with anti-PD-1 therapy demonstrated better tolerability and a trend towards survival benefit (158, 159). A Phase I trial of BLZ945 in solid tumors showed that a subset of patients achieved stable disease, with biomarker analyses indicating reduced TAMs and immune activation (160). A Phase I trial of intratumoral injection of R848 in brain metastasis patients showed good safety and induced immune responses; combination with radiotherapy is being explored (161). A Phase II trial of nivolumab combined with a CSF1R inhibitor in GBM demonstrated an ORR of 15% in the combination group compared to 5% with monotherapy, although larger sample sizes are needed for validation (159). A Phase I trial of an oncolytic virus expressing immunomodulatory molecules preliminarily showed safety and immune activation, with early-phase trials of viruses delivering TRAIL inhibitors underway (118). A Phase II trial of vorinostat combined with TMZ in GBM prolonged PFS but was associated with common hematologic toxicity; biomarkers suggested changes in TAM phenotype (162, 163). Challenges in clinical trials include patient selection, blood-brain barrier penetration, and immune-related toxicity. Biomarkers such as TAM density and polarization status are used to enrich patient populations. Therapeutic strategies targeting TAMs show promise in preclinical and early clinical studies through reprogramming, combination therapies, and new technologies. Future directions include: 1) Selecting therapies based on TAM subtypes (e.g., SPP1+ TAMs) and tumor immune microenvironment characteristics. 2) Optimizing nanoparticles and cellular therapies to enhance brain targeting and reduce off-target effects. 3) Further elucidating interactions between TAMs and neurons and metabolism to develop multi-target strategies. 4) Integrating TAM targeting with existing immune checkpoint inhibitors, radiotherapy, or chemotherapy to maximize synergistic effects. In conclusion, targeting TAMs offers new avenues for brain tumor treatment, but translational challenges must be overcome to achieve clinical benefits.

A critical analysis of these clinical trials reveals key lessons for future TAM-targeted therapy. The modest success of depletion-based strategies (e.g., CSF1R inhibitors) as monotherapies highlights the resilience and redundancy of the immunosuppressive network in brain TME. Conversely, early-phase trials combining TAM modulation (e.g., reprogramming agents) with immune checkpoint inhibitors show a more promising trend, suggesting that altering the functional state of the TME may be more effective than simply reducing macrophage numbers. Furthermore, the variable responses observed in trials emphasize the necessity for patient stratification based on TAM biomarkers​ (e.g., high M2/M1 ratio, GPNMB expression). Future trial designs must move beyond a one-size-fits-all approach and incorporate biomarker-guided enrollment to identify patient populations most likely to benefit.

In recent years, clinical research on BM has been characterized by core trends toward precision, combination, and multimodal integration (**Table 1**). Ongoing clinical trials tailored to different tumor types with brain metastases are steadily advancing, providing key evidence-based support for optimizing clinical treatment strategies. As shown in **Table 1**, current trials primarily focus on the three tumor types with a high incidence of brain metastasis—NSCLC, BC, and melanoma—with a total of 21 studies encompassing phase I–II exploratory trials and comprehensively spanning radiotherapy, targeted therapy, immunotherapy, antibody–drug conjugates (ADCs), and cell therapy. As a focal area in BM research, NSCLC is represented by 10 clinical trials that embody a precision-treatment logic based on molecular subtyping. For patients with EGFR mutations, there are explorations of targeted therapy with osimertinib monotherapy or in combination with bevacizumab (NCT05104281, NCT05948813), as well as post–EGFR inhibitor resistance studies of keynatinib (NCT04824079). For the ROS1 fusion subtype, the oral ROS1 inhibitor JYPO322 offers a new targeted option for patients with brain metastases (NCT06128148). For stage IV NSCLC patients with brain metastases lacking driver mutations, regimens combining PD-1/PD-L1 inhibitors plus chemotherapy with SRT or WBRT (NCT06501391) highlight the growing synergy between immunotherapy and conventional radiotherapy. In addition, platinum-based chemotherapy followed by dual-immunotherapy maintenance with nivolumab plus ipilimumab (NCT05012254) provides a new paradigm of immunotherapy combined with chemotherapy for patients with synchronous brain metastases; this study is currently in active follow-up (**Table 1**).

Clinical trials in breast cancer brain metastases show a distinct subtype-stratified treatment profile, with eight studies tailoring strategies for HER2-positive, HR-positive/HER2-negative, and triple-negative breast cancer (TNBC). In the HER2-positive population, evaluations of pyrotinib monotherapy (NCT06152822), trastuzumab deruxtecan (T-DXd) combined with SRT (NCT06088056), and HER2-targeted CAR-T cell therapy (NCT03696030) expand the application landscape of targeted and cellular therapies. For HR-positive/HER2-negative disease, development centers on the CDK4/6 inhibitor SPH4336 (NCT05872347), providing a new target direction for brain metastases in hormone receptor–positive subtypes. For the hard-to-treat TNBC subtype, the Trop-2–targeting ADC SHR-A1921 combined with bevacizumab (NCT06210438), sacituzumab govitecan monotherapy (NCT06462079), and the combination of adebrelimab plus apatinib plus etoposide (NCT06418594) underscore the potential of ADCs and immunotherapy combined with anti-angiogenic therapy in TNBC brain metastases (**Table 1**).

Research on melanoma brain metastases is concentrated on the BRAF-mutant subtype, with three trials adopting either targeted combinations or a triplet of targeted therapy, immunotherapy, and radiotherapy. The BRAF/MEK inhibitor doublet encorafenib plus binimetinib (NCT03898908) has completed preliminary evaluation, and on this basis, triplet regimens that add pembrolizumab and upfront SRT (NCT04074096, NCT05704933) further strengthen the synergy between local radiotherapy and systemic precision therapy, offering more targeted options for patients with BRAF-mutant melanoma brain metastases (**Table 1**).

Regarding trial phases and status, phase I studies (four in total) primarily assess safety and dose finding, while phase II studies (17 in total) focus on preliminary efficacy, reflecting that innovative BM therapies are still largely in early clinical exploration. Two studies have been completed (NCT03297788, NCT04631029), 11 are actively recruiting, four have completed enrollment and are in follow-up, and three are about to initiate recruitment, forming a continuously advancing research landscape. These trials not only cover the optimization of traditional treatments and innovative combinations but also bring forward emerging modalities such as ADCs and CAR-T cell therapy. They generally emphasize stratification by genomic status, tumor subtype, and prior treatment history, fully reflecting the central role of precision medicine in the treatment of brain metastases and providing important support for subsequent clinical translation and guideline updates.

## 7 Challenges and future directions

Research on TAMs has made significant strides in brain metastasis and primary brain tumors, yet multiple challenges hinder the full translation and clinical application of therapeutic strategies. This section systematically outlines the primary challenges in current TAM research, such as heterogeneity, technical limitations, and barriers to personalized medicine. It also envisions future directions, including the application of new technologies and precision medicine strategies. The focus will cover challenges of heterogeneity, the cutting-edge of technology, and personalized medicine, providing a framework for future research.

### 7.1 The therapeutic imperative of TAM heterogeneity: implications for precision medicine

The profound heterogeneity of TAMs is not merely a biological curiosity but a central determinant of therapeutic efficacy and a major challenge in brain tumor therapy. The composition, origin, and functional states of TAM subsets vary significantly across different cancer types (e.g., breast cancer vs. lung cancer) and even within individual tumors. This variability means that a one-size-fits-all approach to targeting TAMs is destined to fail. Instead, therapeutic strategies must be tailored based on the specific TAM landscape, which necessitates a deeper understanding of how heterogeneity dictates treatment selection. The following sections dissect this heterogeneity from three critical perspectives: cancer-type specificity, cellular origin, and spatiotemporal dynamics.

The heterogeneity of TAMs is one of the primary challenges in treating brain tumors, as the composition and functional states of their subtypes exhibit significant differences across various cancer types (e.g., breast cancer, lung cancer, and glioblastoma), affecting treatment responses and prognosis. This heterogeneity arises from multiple factors. Firstly, the origin and recruitment of TAMs vary. In breast cancer brain metastasis, TAMs primarily originate from peripheral monocytes, recruited via the CCL2/CCR2 axis, and are enriched with M2-like subtypes (e.g., CD206+ TAMs), promoting angiogenesis and immune suppression (4). In contrast, TAMs in lung cancer brain metastasis include a higher proportion of microglia-derived cells, which mediate blood-brain barrier disruption through MMP9, enhancing tumor cell infiltration (4). scRNA-seq data show that TAMs in breast cancer brain metastasis highly express SPP1 and ARG1 (164), whereas TAMs in lung cancer brain metastasis upregulate CX3CR1 and TREM2, reflecting tissue-specific adaptations (165, 166). Secondly, the functional states of TAMs are variable. In different cancer types, the polarization status and immunoregulatory functions of TAMs differ. For example, in GBM, TAMs tend towards M2 polarization, suppressing T cell functions through IL-10 and TGF-β (167); whereas in melanoma brain metastasis, TAMs exhibit a mixed M1/M2 phenotype and can shift towards M1 in response to IFN-γ (4). This functional variability makes single-target strategies (e.g., CSF1R inhibitors) ineffective in some tumors due to compensatory mechanisms. Finally, the heterogeneity of TAMs is influenced by various drivers within the microenvironment. The unique characteristics of the TME, such as hypoxia and metabolite accumulation, further exacerbate heterogeneity. In breast cancer brain metastasis, hypoxia induces VEGF expression in TAMs through HIF-1α, promoting angiogenesis (168); while in GBM, neuronal activity (e.g., BDNF signaling) drives TAMs to form synapse-like connections with tumor cells, enhancing colonization (169). Single-cell analyses reveal that TAM subpopulations (e.g., lipid-associated macrophages) exhibit different spatial distributions across cancer types, impacting local immune responses (59). The challenge of heterogeneity underscores the need for cancer-type-specific TAM-targeting strategies, based on detailed molecular profiling.

This cancer-type specificity demands distinct therapeutic approaches. For instance, in breast cancer BM where peripheral recruitment via CCL2/CCR2 is dominant, CCR2 inhibitors​ might be particularly effective (4). In contrast, for lung cancer BM with significant microglial contribution and MMP9-mediated BBB disruption, strategies that modulate microglial activation​ or inhibit MMP9 could be more relevant (4). This underscores the need for preclinical models and clinical trials that are disease-specific. The cellular origin of TAMs influences their susceptibility to therapy. For example, peripherally derived macrophages may be more effectively depleted by CSF1R inhibitors​ due to their dependence on CSF1 for survival. In contrast, resident microglia might be more resilient to depletion but could be better targets for reprogramming strategies​ (e.g., TLR agonists) that modulate their innate immune functions. Therefore, profiling the origin ratio in a patient’s tumor could guide the choice between depletion and reprogramming strategies. The spatial and functional plasticity of TAMs means that therapeutic targeting must consider the tumor’s anatomical and metabolic context. Hypoxia-specific TAM subpopulations (e.g., LAMs) could be targeted with HIF inhibitors​ or drugs disrupting lipid metabolism (168). Meanwhile, TAMs promoting vessel co-option in perivascular niches might require localized drug delivery​ strategies. This spatial heterogeneity argues against systemic administration of broad-acting agents and in favor of targeted or combination approaches that address multiple TAM niches simultaneously.

A major challenge in targeting TAMs therapeutically is their profound functional heterogeneity. A ‘one-size-fits-all’ approach, such as global CSF1R inhibition to deplete TAMs, may fail because it affects both pro-tumoral and potentially anti-tumoral subsets. Future strategies must consider the specific TAM subpopulation driving disease progression in a given patient. For instance, therapies targeting TREM2+ LAMs​ might be ideal for overcoming immunosuppression in hypoxic niches, while strategies aimed at interferon-primed TAMs​ could be designed to boost their inherent anti-tumor functions.

Future therapeutic development must prioritize the functional subtyping of TAMs. The goal is to move from a one-size-fits-all approach to a personalized strategy where treatment is selected based on the predominant TAM-driven pathology in an individual patient—whether it is angiogenesis, immunosuppression, or metabolic suppression.

### 7.2 Technological frontiers: single-cell sequencing, spatial transcriptomics, and microbiome analysis reveal new targets

The application of advanced technologies is gradually overcoming the challenges posed by TAM heterogeneity, providing high-resolution insights that help identify new therapeutic targets. scRNA-seq allows for the dissection of the transcriptomic diversity of TAMs at the single-cell level. In GBM and brain metastases, scRNA-seq has identified previously unknown TAM subpopulations, such as TREM2+ lipid-associated macrophages (LAMs) and CX3CR1+ patrolling macrophages, which are associated with immune suppression and tumor progression (59). For instance, scRNA-seq analyses have shown the upregulation of microbial response genes in TAMs, suggesting that bacterial elements might regulate TAM functions (170). This technology also reveals the state transition trajectories of TAMs, such as the plasticity from M2 to M1 polarization, providing a time window for reprogramming therapies. Moreover, spatial transcriptomics technologies (such as GeoMX and Visium) integrate morphological and molecular data to reveal the spatial distribution and cellular interactions of TAMs within the TME. In brain tumors, spatial analysis has shown that TAMs cluster around blood vessels and at the tumor-brain interface, correlating with ECM remodeling and the formation of immune-privileged zones (171). Spatial transcriptomics has linked specific TAM subpopulations to microbial signaling hotspots, suggesting that microbial elements might locally modulate immune responses. This spatial resolution aids in identifying region-specific targets, such as TAM subpopulations associated with vascular co-selection. Additionally, microbiome studies utilizing 16S rRNA sequencing and metagenomics explore the influence of microbial elements (e.g., bacteria) on TAM regulation within the TME. Microbiome analyses have detected intracranial bacterial signals and developed rigorous workflows (including FISH and sequencing) to confirm their presence. Microbial elements, such as LPS, might activate TAMs through TLR signaling, influencing their polarization state (172). However, microbiome analyses face challenges due to low biomass, necessitating cautious interpretation. These technological frontiers not only deepen our understanding of TAM biology but also reveal new targets, such as microbial-TAM interaction pathways or spatially restricted immune checkpoints. In the future, multi-omics integration (e.g., scRNA-seq with proteomics) will provide a more comprehensive perspective.

### 7.3 Personalized medicine: patient stratification based on TAM biomarkers

Personalized medicine aims to stratify patients based on TAM biomarkers to optimize therapeutic choices and improve prognoses (**Figure 2**). Glycoprotein non-metastatic melanoma protein B (GPNMB) is highly expressed in M2-like TAMs and is associated with immunosuppression and tumor progression (173). In GBM, GPNMB+ TAMs are enriched in hypoxic regions and promote therapeutic resistance by modulating lipid metabolism (173, 174). Clinical trial data indicate that GPNMB levels correlate with responses to anti-CSF1R therapy, suggesting that patients with high GPNMB expression may benefit from combined treatments (175). TRAIL, expressed by TAMs and astrocytes, drives immune evasion by inducing T cell apoptosis. TRAIL+ astrocytes are associated with GBM recurrence and shorter survival; inhibiting TRAIL signaling enhances T cell function and therapeutic response (118, 176). TRAIL expression can serve as a stratification biomarker to guide TRAIL-targeted therapies, such as oncolytic virus-delivered inhibitors (118). Additionally, biomarkers including CD163 (an M2 marker), PD-L1 (an immune checkpoint), and SPP1 (a lipid-associated marker) have been validated in scRNA-seq and IHC analyses, correlating with TAM subtypes and clinical outcomes (118). Therefore, we can establish personalized medical strategies that include: 1) Designing enrichment trials based on TAM biomarkers (such as GPNMB or TRAIL) to select patient subgroups likely to respond to specific therapies (e.g., CSF1R inhibitors or immunotherapy combinations). 2) Tracking TAM status changes through liquid biopsies or imaging to assess therapeutic response and resistance. For instance, PET imaging targeting TAM markers (such as CD206) is under exploration. 3) Combining treatments based on TAM subtypes, such as reprogramming agents targeting M2 TAMs with immune checkpoint inhibitors, to maximize synergistic effects. However, these strategies face various challenges, including biomarker validation, standardization of detection methods, and costs. Technological advancements, such as AI-assisted image analysis, are gradually alleviating these issues.

### 7.4 Spatial and temporal dynamics of TAMs in brain metastasis

The therapeutic targeting of TAMs is further complicated by their profound spatial and temporal heterogeneity. Spatially, distinct TAM subpopulations occupy and functionally shape specific anatomical niches within the brain metastatic lesion. For instance, the hypoxic tumor core is often enriched with LAMs that promote immunosuppression, while the invasive margin and perivascular areas harbor TAMs that facilitate cancer cell invasion and vessel co-option. This spatial compartmentalization means that a therapeutic agent must not only hit the right molecular target but also reach the relevant functional niche to be effective. Temporally, the TAM landscape is not static but evolves dynamically during disease progression and in response to therapies. The initial colonization phase, dominated by resident microglia, differs significantly from the advanced metastatic stage, which is characterized by an influx of monocyte-derived macrophages. Furthermore, treatments like radiotherapy or chemotherapy can dramatically reshape the TAM repertoire, for example, by inducing a pro-inflammatory response that may later resolve into a more immunosuppressive state contributing to treatment resistance. Therefore, a successful therapeutic strategy must account for this spatiotemporal complexity, requiring biomarkers to map the dominant TAM subsets at a given time and location, and adaptable combination regimens that target multiple TAM functions simultaneously or sequentially.

## 8 Conclusion and future perspectives

Research on TAMs in brain tumors faces challenges related to heterogeneity, technical limitations, and personalized medicine. However, emerging technologies such as single-cell and spatial genomics, along with biomarkers like GPNMB and TRAIL, offer potential solutions. Future directions should integrate scRNA-seq, spatial transcriptomics, and microbiomics to construct a comprehensive TAMs atlas and identify actionable targets. Additionally, utilizing organoids and humanized mouse models to simulate TAM heterogeneity and test personalized therapies will be crucial. Designing biomarker-driven clinical trials will validate the efficacy of TAM-targeted strategies in specific patient subgroups. In summary, overcoming these challenges will advance TAM-targeted therapies towards precision medicine, ultimately improving the prognosis of brain tumor patients. Future studies should leverage lineage-tracing techniques to delineate the relative contributions of TAMs from different origins to therapeutic resistance, thereby informing more targeted combination therapies.
