# Correction: Diversity and function of tumor-associated macrophages in brain metastases: mechanisms and therapeutic prospects

**DOI:** 10.3389/fimmu.2026.1820405

**Published:** 2026-08-07

**Authors:** Yingping Ma, Hongyu Wang, Xinman Dou, Qiang Li

**Affiliations:** 1 Department of Neurosurgery, The Second Hospital of Lanzhou University, , Lanzhou, China; 2 Nursing Department, The Second Hospital of Lanzhou University (The Second Clinical Medical College), Lanzhou, China

**Keywords:** brain metastasis, immunosuppression, immunotherapy, tumor microenvironment, tumor-associated macrophages

Following publication of this article, concerns were raised regarding the use of modified, copyright-restricted material in **Figure 1**. **Figure 1** was modified from **Figure 2** of Schreurs et al., 2024 (1), originally published under an all-rights-reserved copyright. To ensure compliance with the license terms, **Figure 1** has been replaced with a new, original image.

**Figure 1:**
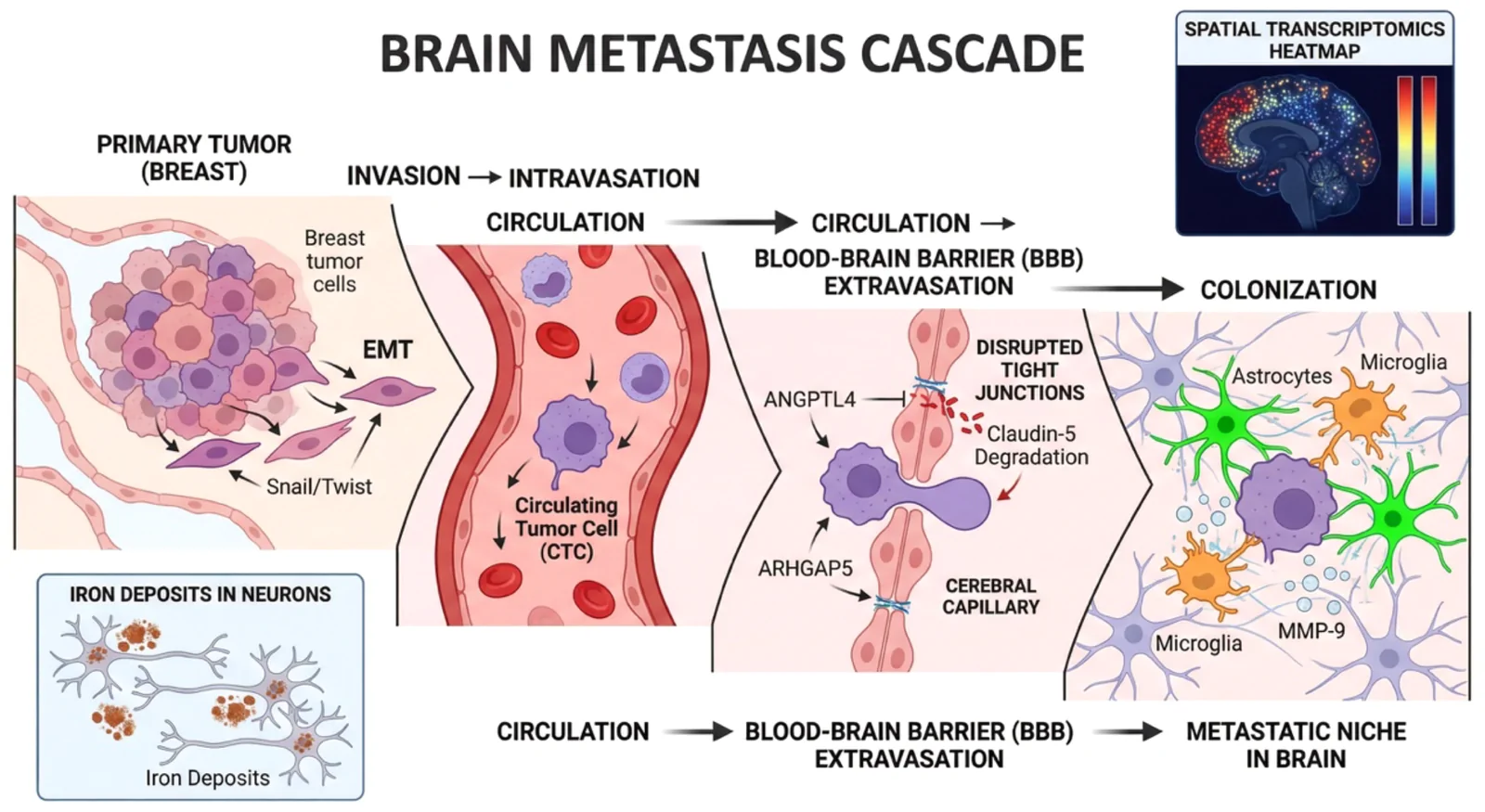
Mechanistic cascade of breast cancer brain metastasis.

Directional arrows depict the metastatic progression from primary tumor invasion through intravasation, circulation, and culminating in colonization. Within the breast tissue microenvironment, transcription factors Snail and Twist drive epithelial-mesenchymal transition to facilitate tumor cell detachment. Circulating tumor cells (CTCs) shown in purple disrupt the blood-brain barrier via degradation of Claudin-5 tight junctions and activation of the ANGPTL4/ARHGAP5 pathway. In the established brain metastatic niche, star-shaped astrocytes colored green interacts with amoeboid orange microglia secreting MMP-9 to remodel extracellular matrix, while rust-colored iron deposits accumulate within neurons.

The original version of this article has been updated.
